# The Impact of Head Position on Neurological and Histopathological Outcome Following Controlled Automated Reperfusion of the Whole Body (CARL) in a Pig Model

**DOI:** 10.3390/jcm12227054

**Published:** 2023-11-13

**Authors:** Domagoj Damjanovic, Jan-Steffen Pooth, Yechi Liu, Fabienne Frensch, Martin Wolkewitz, Joerg Haberstroh, Soroush Doostkam, Heidi Ramona Cristina Schmitz, Katharina Foerster, Itumeleng Taunyane, Tabea Neubert, Christian Scherer, Patric Diel, Christoph Benk, Friedhelm Beyersdorf, Georg Trummer

**Affiliations:** 1 Department of Cardiovascular Surgery, University Medical Center Freiburg, Faculty of Medicine, University of Freiburg, Hugstetter Str. 55, D-79106 Freiburg, Germany; 2 Department of Emergency Medicine, University Medical Center Freiburg, Faculty of Medicine, University of Freiburg, D-79106 Freiburg, Germany; 3 Institute of Medical Biometry and Statistics, Division Methods in Clinical Epidemiology, Faculty of Medicine and Medical Center, University of Freiburg, D-79104 Freiburg, Germany; 4 Experimental Surgery, Center for Experimental Models and Transgenic Service, University Medical Center Freiburg, Faculty of Medicine, University of Freiburg, Breisacher Str. 66, D-79106 Freiburg, Germany; 5 Institute of Neuropathology, University Medical Center Freiburg, Faculty of Medicine, University of Freiburg, Breisacherstr. 64, D-79106 Freiburg, Germany; 6 Center for Experimental Models and Transgenic Service, University Medical Center Freiburg, Faculty of Medicine, University of Freiburg, Stefan-Meier-Str. 17, D-79104 Freiburg, Germany

**Keywords:** ischemia-reperfusion, extracorporeal life support, venous return, porcine cardiac arrest model, bundle of care, targeted CPR, eCPR, VA-ECMO, pathophysiology of cardiac arrest

## Abstract

**Introduction:** Based on extracorporeal circulation, targeted reperfusion strategies have been developed to improve survival and neurologic recovery in refractory cardiac arrest: Controlled Automated Reperfusion of the whoLe Body (CARL). Furthermore, animal and human cadaver studies have shown beneficial effects on cerebral pressure due to head elevation during conventional cardiopulmonary resuscitation. Our aim was to evaluate the impact of head elevation on survival, neurologic recovery and histopathologic outcome in addition to CARL in an animal model. **Methods:** After 20 min of ventricular fibrillation, 46 domestic pigs underwent CARL, including high, pulsatile extracorporeal blood flow, pH–stat acid–base management, priming with a colloid, mannitol and citrate, targeted oxygen, carbon dioxide and blood pressure management, rapid cooling and slow rewarming. N = 25 were head-up (HUP) during CARL, and N = 21 were supine (SUP). After weaning from ECC, the pigs were extubated and followed up in the animal care facility for up to seven days. Neuronal density was evaluated in neurohistopathology. **Results:** More animals in the HUP group survived and achieved a favorable neurological recovery, 21/25 (84%) versus 6/21 (29%) in the SUP group. Head positioning was an independent factor in neurologically favorable survival (*p* < 0.00012). Neurohistopathology showed no significant structural differences between HUP and SUP. Distinct, partly transient clinical neurologic deficits were blindness and ataxia. **Conclusions:** Head elevation during CARL after 20 min of cardiac arrest independently improved survival and neurologic outcome in pigs. Clinical follow-up revealed transient neurologic deficits potentially attributable to functions localized in the posterior perfusion area, whereas histopathologic findings did not show corresponding differences between the groups. A possible explanation of our findings may be venous congestion and edema as modifiable contributing factors of neurologic injury following prolonged cardiac arrest.

## 1. Introduction

Neurologically favorable survival in cardiac arrest (CA) is dramatically low despite advanced life support and high quality cardiopulmonary resuscitation (CPR) [1,2,3,4]. Therapy bundles and a system-based approach rather than stand-alone interventions might improve these outcomes [5,6,7,8,9,10].

On the *tissue and cellular level*, one of the main therapeutic objectives in such bundles is in the mitigation of ischemia-reperfusion injury (IRI). Controlled Automated Reperfusion of the whoLe body (CARL) is an individualized therapy bundle based on extracorporeal circulation that is designed to reduce IRI. With CARL, favorable neurologic recovery has been be achieved in a porcine cardiac arrest model, even after 20 min of normothermic cardiac arrest [11,12].

From a *macro-hemodynamic perspective*, traditionally, the restoration of arterial perfusion of organs and tissues has been considered the primary aim during cardiac arrest. More recent resuscitation research, however, suggests that cerebral venous drainage and venous return to the central circulation are equally as important. The assumption that venous congestion during cardiac arrest adds to neuronal damage has led to the development and marketing of devices designed to increase venous return during CPR, thereby improving survival and neurologic outcome.

Elevated positioning of the head is another simple intervention to enhance cerebral venous drainage. In neuro-critical care, as well as post-resuscitation care, it is an established measure for prophylaxis and therapy of increased intracranial pressure (ICP) [13]. Following the concept of *intra-arrest* venous congestion as an important pathophysiologic contributor to bad neurologic outcome, head elevation has been applied *during* conventional CPR and was shown to reduce ICP in an animal model [14,15].

It has been combined with other methods for enhancing venous return in further studies [16]. However, to our best knowledge, it has not yet been studied in conjunction with extracorporeal circulation.

The aim of our study was, therefore, to evaluate the impact of head elevation on survival, neurologic recovery and neuronal loss in combination with CARL therapy.

## 2. Methods

Animal experiments were approved by the local ethics committee (Freiburg, Germany, approval number G-15/148) and performed in accordance with the rules and regulations of the German animal protection law and the animal care guidelines of the European Community (2010/63/EU). The ARRIVE Checklist is added as Supplement S1.

**Figure 1:**
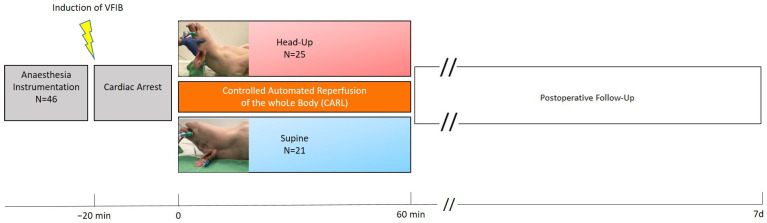
Timeline of the experiment. VFIB: ventricular fibrillation.

The basic protocol of this chronic porcine cardiac arrest model has already been published in detail [11]. Figure 1 depicts the timeline of the experiment. This study was conducted as a pooled analysis. Experiments were carried out in an open-label parallel-group design. The group allocation was non-randomized, block-wise.

Twenty-eight juvenile domestic pigs were anesthetized, and ventricular fibrillation was induced. Before anesthesia, they were fasted overnight with free access to water. After 20 min of normothermic CA, CARL was started. It comprises a targeted reperfusion therapy bundle based on arteriovenous extracorporeal membrane oxygenation. This, in brief, consists of high, pulsatile extracorporeal blood flow, pH–stat acid–base management, priming with a colloid (20% human albumin (Albiomin 200 g/L, Biotest Pharma GmbH, Dreieich, Germany; 200 g/L, Baxalta Deutschland GmbH, Unterschleissheim, Germany; or 200 g/L, Octapharma GmbH, Langenfeld, Germany, respectively, depending on availability)) or Gelatin-Polysuccinate (Gelafundin 40 g/L, B. Braun Melsungen AG, Melsungen, Germany)), mannitol and citrate [17], tight oxygen and carbon dioxide control, rapid cooling and slow rewarming before weaning off the extracorporeal circulation and ECC [11]. Instead of electric defibrillation, ventricular fibrillation was preferably terminated through cardioplegic potassium bolus [18]. The hyperosmolar priming solution has a neuroprotective potential, whereas pharmacologic defibrillation via secondary cardioplegia aims at cardioprotection through avoidance of the harmful impact of serial electric defibrillations on cardiac tissue. A detailed overview of the CARL therapy, including rationales for its single components, as well as images and an explanatory video, can be found in Ref. [19].

Head elevation was conducted using a standardized pillow (towel roll), with additional regular inclinations for up to 90°. This is depicted in Supplement S3/Supplementary Figure S1. An additional 18 animals from historic groups with an otherwise identical intervention protocol were added for pooled analysis regarding head positioning.

Overall, 25/46 pigs underwent CARL with head-up positioning HUP (sixteen current, nine historic sample), and the other 21 were positioned supine (SUP) (twelve current, nine historic sample).

After completion of the reperfusion protocol, animals were weaned off the ECC and the ventilator and subsequently extubated. Follow-up in the CEMT facility was conducted for up to seven days. Postoperative care was conducted following a standard protocol. On postoperative day (POD) 3, interim analysis and scoring were performed in accordance with this protocol. The assessors were trained members of the research group, supervised by a veterinarian, not blinded to the group allocation. The neurologic outcome was characterized using a species-specific neurologic deficit score. A score of 500 denotes brain death, whereas animals with NDS < 50 were able to stand up independently, walk without help and eat on their own. A score < 50 was, therefore, defined as a surrogate of favorable neurologic outcome or significant potential for rehabilitation, respectively. Upon clinical suspicion, distinct neurologic deficits were evaluated separately. Following regulatory requirements, a prespecified set of criteria was applied for early termination of the survival experiment (NDS > 200 after 24 h or >120 after 48 h, suspicion of vegetative state, inhumane suffering, inadequate non-neurologic recovery or poor general health status) [11].

After completion of clinical follow-up and euthanasia, the porcine brains were retrieved for neuro–histopathologic workup, for which hematoxylin–eosin staining was used. For this purpose, a frontal craniotomy was performed. After transecting at the level of the medulla oblongata, brains were extracted as a whole and immediately immersed in formaldehyde 4%. Following one week of immersion, the brains were dissected, and 5 mm samples of the frontal lobe, cerebellar vermis and hippocampus were obtained, respectively. In identifying those areas as our regions of interest, we sought to procure a representation of both the territories of the anterior and occipital cerebral blood supply, respectively. Furthermore, the hippocampal region has been identified as one of the most susceptible regions for cerebral ischemia-reperfusion injury following cardiac arrest [20]. In the next step, the samples were dehydrated and embedded in paraffin. 3µm slices of the samples underwent hematoxylin–eosin staining. Consequently, slides were masked and digitalized, and representative areas were examined in terms of neuron count to determine neuron density. Only neurons with a clearly identifiable nucleus were considered to be viable and have been included in the total count.

### Statistical Methods

Continuous baseline characteristics were compared between the groups (HUP and SUP) using the Kruskal–Wallis rank sum test. Kaplan–Meier survival curves were calculated to compare survival up to 7 days between the groups. Regarding NDS, linear mixed models (see Brown and Prescott, 1999) have been fitted with a random intercept (subject = pig). The continuous response variable “NDS” is modeled as a linear function of time (continuous) and group (HUP or SUP), including an interaction term. We further included all baseline characteristics if they showed significant variations between the groups. All computations were performed using the statistical software R system (version 4.3.0). They are added as Supplement S2.

## 3. Results

Baseline characteristics of the animals are shown in Table 1.

- Intra-operative course

After starting reperfusion, electric defibrillation was inevitable to terminate VF in three pigs. In all other animals, secondary cardioplegia through a potassium bolus was sufficient for rhythm conversion. Intraoperative monitoring revealed no systematic differences between the animals with good or bad neurologic outcome. Table 2 shows mean arterial blood pressure values during the experiment, i.e., at baseline and during controlled reperfusion. Blood flow rates achieved were high, as targeted; mean cardiac index during the first 15 min of reperfusion was ≥5.0 L/min/m^2^ in all but two animals (4.87 and 4.95 L/min/m^2^).

- Survival, neurologic outcome and clinical status

**Figure 2:**
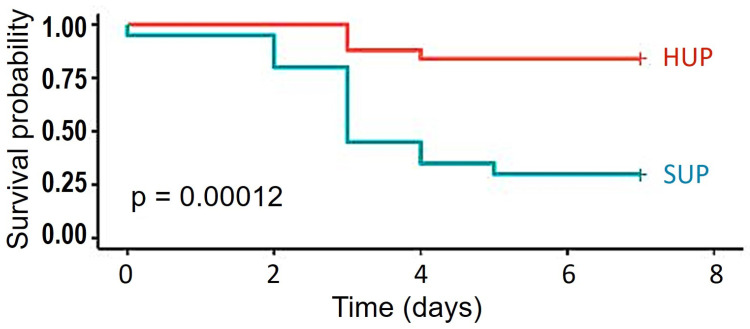
Survival probability following the experiments, in correlation to head positioning. HUP: head-up, SUP, supine.

**Figure 3:**
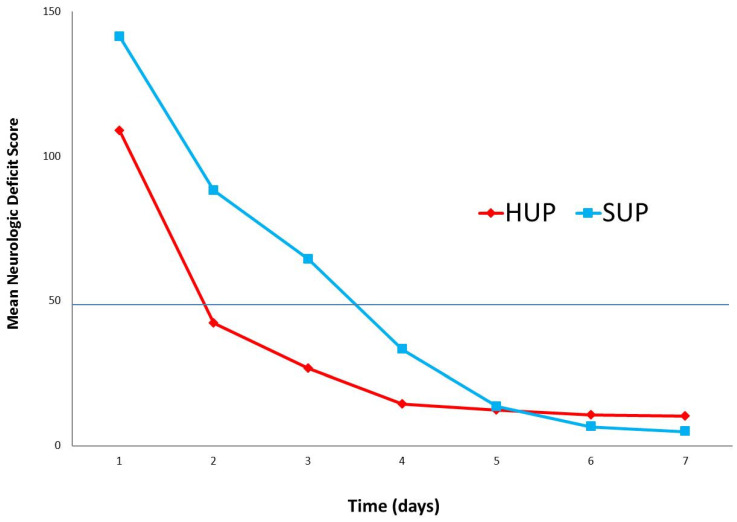
Mean neurologic deficit score during the postoperative course. HUP: Head-up, SUP, supine.

Forty-five out of forty-six pigs were successfully weaned off ECC after 1 h of CARL. One animal could not be weaned off ECC due to refractory hemodynamic failure, and was excluded from further analysis. In all others, extubation was possible after 3 h from the start of the intervention. In Table 2, the time course of neuron-specific enolase as a surrogate marker of neurologic injury can be appreciated. No significant differences were found between the HUP and the SUP group. NSE did, however, predict worse outcomes in the NDS score, i.e., animals that did not achieve the predefined threshold of NDS = 50 for good neurologic outcome on day 1 and day 7, respectively (*p* = 0.04787 and *p* = 0.003094). Figure 2 shows a higher survival probability in the head-up group compared to the supine group over the follow-up period of one week, displayed as a Kaplan–Meier survival curve. Figure 3 depicts the development of the NDS values over time, with a threshold of 50 points considered to be a good recovery. Starting with scores between 100 and 150 on the first day after the intervention, more animals in the head-up group achieved neurological recovery compared to supine positioning: 21/25 (84%) vs. 6/20 (30%). During clinical follow-up, predominant clinical problems were transient blindness and ataxia in otherwise alert animals, most of whom started eating and drinking early on in the postoperative course. After adjusting for different confounders, such as plasma hemoglobin, priming composition (gelatin or albumin, see Table 3), priming osmolality or sodium concentration, head positioning remained an independent factor in neurologically favorable survival (*p* < 0.00012).

### Neurohistopathology

**Figure 4:**
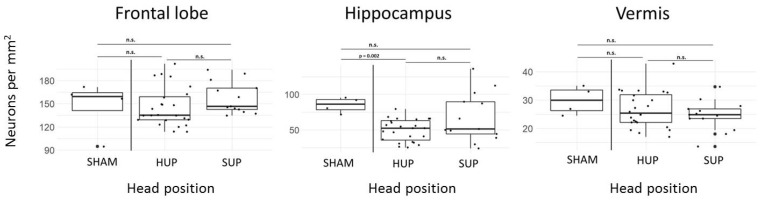
Neurohistopathologic analysis of total number of neurons per square millimeter in frontal lobe, hippocampus and vermis. Boxplots (median, interquartile range) are shown in correlation to head position. SHAM: sham animals. HUP: head-Up; SUP: supine. ns: not significant. Samples were available for workup in N = 24 in the HUP and N = 12 in the SUP group. Sham animal controls did not undergo ischemia, CPR or extracorporeal circulation. Head-up animals had significantly lower neuron counts in the hippocampus when compared to controls. All other differences were not statistically significant.

Figure 4 shows a neurohistopathologic evaluation of neuron density in the cerebellum, frontal cortex and hippocampus in correlation to the head position. Samples were available for workup in *n* = 24 in the HUP and *n* = 12 in the SUP group. Four sham animals did not undergo ischemia, CPR or extracorporeal circulation. A significantly lower neuron count was found in the hippocampus of head-up animals compared to controls, while all other differences were not statistically significant.

## 4. Discussion

In this experimental large animal cardiac arrest study, we showed that (1) in conjunction with the CARL therapy bundle, elevation and repeated inclination of the head after 20 min of normothermic cardiac arrest improved survival and neurologic recovery compared to supine positioning, but (2) without corresponding damage patterns in histopathology. To our best knowledge, this is the first translational study to combine elevation of the head with extracorporeal reperfusion following prolonged cardiac arrest. Furthermore, post-interventional follow-up for several days allowed for clinical assessment, characterization of neurologic deficits and dynamics of recovery. The majority of translational resuscitation studies are conducted as physiology-centered terminal experiments, without awakening the animals after the intervention, and hence without the opportunity for neurologic scoring and follow-up. Instead, data on trajectories of decreased intracranial pressure, improved cerebral perfusion or metabolism, respectively, have been reported in several studies on head elevation during CPR [14,15,21,22,23,24]. Two systematic reviews and meta-analyses provide a further synopsis [25,26]. The results of studies using a postoperative follow-up are conflicting: One randomized study by Park et al. found lower ROSC-rates and 24 h survival in pigs undergoing CPR after 15 min of untreated cardiac arrest, with the whole body tilted 30 degrees head-up. Neurologic outcomes were not reported [27]. More recently, Moore et al. showed an improved neurologic survival with HUP in one study with Yorkshire pigs in a 24 h follow-up period after 10 min of untreated cardiac arrest, followed by 9 min of CPR [16]. Therefore, the results of our study may complement the predominantly physiologic data at least to some extent, providing information on the most robust and most desired target outcome of CPR, the neurologically favorable outcome.

The neurologic deficit score we used for that purpose is a standardized instrument that supports comparability with other translational studies in resuscitation research. Regarding the severity of functional impairment, mean deficit scores under 150 are considerably low already after 24–48 h, given the major insult of 20 min no-flow-time. The score comprises different neuro-functional aspects. However, the clinical constellations we observed, i.e., ataxia and transient blindness, were not fully covered. This is further discussed in the Section 4.2.

The enhancement of venous drainage from the brain to the thorax, which has been reported with conventional CPR, might be a possible explanation for this observation in our study as well, although it was not directly measured.

### 4.1. Structural Correlation of Damage Patterns

Traditional veterinary anatomical studies, as well as recent functional magnetic resonance imaging studies, show structural representation of visual functions and posturing in the posterior cerebral and cerebellar areas. Lesions herein are consistent with visual impairment and postural problems seen in the postoperative course in many of the animals. In the supine position, intravascular *stasis* during arrest is likely to predominate in the posterior areas. An impaired *venous* drainage through the prominent prevertebral venous plexus and *venous* congestion in the sinus sigmoideus, transversus and the pronounced confluens sinuum will gradually affect adjacent areas, that is, the abovementioned posterior parts again. Stasis and congestion will result in tissue edema. This might explain the transient nature of visual loss, with regression of the edema leading to the regaining of sight. An MRI study on a Yucatan pig by Habib et al. further illustrates the relation between porcine cerebral venous vasculature and possible ways of drainage [28].

The structural damage patterns (loss of neurons) actually seen in the histopathologic workup of our sample do not match the clinical findings. These were (1) obvious neurologic damage in all animals immediately after profound ischemia-reperfusion injury and (2) significantly better functional recovery in the head-up group, despite its increased loss of hippocampal neurons, when compared to the sham and supine groups. In the literature, the hippocampus; basal ganglia; frontal, parietal, temporal and occipital cortices; as well as the cerebellar cortex, were found to be most susceptible to ischemia and hypoxia, respectively [18]. Methodological limitations in recovery and workup of brain tissue specimens may have contributed to these conflicting findings (see Section 4.2.). Another possible explanation is that neurologic damage was not only transient from a clinical perspective, but also in terms of reversible tissue edema. This edema cannot be detected by the histopathological methods used herein, and might have been more pronounced and less regredient in the supine group. The increased hippocampal neuron loss observed in the tissue of HUP animals obviously had less of a clinically relevant impact on the neuro-functional status and recovery than other histopathologically inapparent changes. Furthermore, neuron-specific enolase (NSE) concentrations did not differ between the groups; they did, however, predict worse neurologic outcome. As a serum marker of neuronal injury, NSE was only moderately elevated. Notably, the molecule is also contained in erythrocytes and hence can be elevated due to hemolysis, e.g., when extracorporeal circulation is applied. Due to the mechanism of experimental injury itself and the extracorporeal reperfusion with a dual blood pump configuration, respectively, higher NSE-values could have been expected. In humans, the prognostic utility of NSE in predicting poor outcomes after cardiac arrest has been studied extensively. Reported cut-off values range between 33 and 120 μg/L [28]. Following extracorporeal cardiopulmonary resuscitation, Haertel et al. found NSE serum levels of >55.9 µg/L after 48 h post-arrest to be predictive of worse neurologic outcomes [29]. In pigs, Vammen et al. described mean NSE values below 10 µg/L up to 48 h following 11 min of untreated cardiac arrest, with markedly higher levels in more severely affected animals [30]. NSE values are time-dependent and prone to confounding [31]. Therefore, current international guidelines for post-resuscitation care suggest that NSE should only be used in conjunction with other prognostic tests because “no single test has sufficient specificity to eliminate false positives” [28]. In previously published work from our group by Foerster et al., even after 20 min of untreated cardiac arrest, low levels of 4.6 and 1.5 µg/L in a hypothermic group versus 5.6 µg/L and 4.3 µg/L in a normothermic group were found on day 1 and day 7 of the follow-up period, respectively [12]. According to Taunyane et al., an up to twenty-fold increase to 7.95 µg/L at the end of the experiment compared to the baseline was only seen in an uncontrolled reperfusion group with 15 min of cardiac arrest, 10 min conventional CPR and 60 min of normothermic reperfusion [11].

The clinical course in our study might support the veno-congestive edema hypothesis, as would the benefit of head elevation ex juvantibus. In summary, intraoperative physiology, structural changes, serum markers and clinical outcomes can differ significantly. In isolation, the acute setting obviously has limited predictive value, which underlines the need for clinical correlation and long-term neurologic follow-up in translational resuscitation research.

### 4.2. Limitations

Due to the complexity of the experimental model and the therapy bundle applied, it is possible that not all confounders were known or controlled for, respectively. Furthermore, we did not directly measure cerebral tissue perfusion or intracranial pressure in real time to prove the congestion hypothesis. Anticoagulation was necessary during extracorporeal circulation. The concurrent intracranial bleeding risk following invasive ICP measurement was deemed unacceptably high in this experimental setting. In the histopathologic workup, there is an increased dropout-rate of specimens in the supine group, which is in part due to the retrospective nature of the analysis. As mentioned above, a historic sample of the study population, which underwent the same experimental procedure, has been added for pooled analysis. This included the post hoc consideration of historic histopathologic samples, some of which were no longer available. From an ethical standpoint in translational animal research, we decided to use all available information and, hence, the biggest possible number of samples instead of eliminating samples from the HUP group to achieve a 1:1 ratio.

The only moderately increased NSE levels we observed are consistent with previous findings in this research model by Foerster et al. [12] and Taunyane et al. [11]. The best test performance of NSE has been described at 48 and 72 h post-cardiac arrest, with “only limited evidence” for the period after 72 h [28]. Following our protocol, blood sampling was only possible on day 1 and day 7 or at end of the experiment, respectively. Hence, we might have missed the relevant time window and true peak levels.

The NDS score used to formalize clinical observations cannot account for specific constellations, as found in our study. It is an inherent limitation of the score and our mode; due to the interdependence between the visual system and the equilibrium sense, visual impairment directly affects postural control, even if there is no further damage to the vestibular or cerebellar apparatus itself. Thus, transient blindness and gait disturbance may lead to an overestimation of neurologic damage in otherwise alert and physiologically interacting animals. This overestimation could have prevented some of these animals with significant recovery potential from crossing the pre-defined 72 h cut-off point, therefore being sacrificed early. On the other hand, the NDS does not cover aspects of general well-being or non-neurological injury, which might induce suffering and trigger premature sacrifice in translational survival experiments. We did not use formal scoring systems for general well-being; however, we are not aware of major non-neurological impairments in this study sample.

### 4.3. Transferability

The transferability of these translational research results to resuscitation in humans might be limited by the different vascular anatomy, especially regarding venous drainage of the brain. However, recent experimental data suggest a potentially beneficial role in human patients. A first clinical study involving a bundled head-up/torso-up approach by Pepe et al. showed a doubling of resuscitation rates, i.e., return of spontaneous circulation on hospital arrival after introducing the bundle [32]. Rates of neurologic recovery remained proportional, however, and the bundle also included other adjustments of the CPR-management, i.e., using the Impedance Threshold Device (ITD) and Active Compression-Decompression Device (ACD), as well as performing pit crew CPR. A more recent observational study by Moore et al. displayed no difference in primary or secondary endpoints among 860 patients with Out-of-Hospital Cardiac Arrest (OHCA) undergoing conventional CPR versus 222 propensity-score-matched OHCA patients with automated sequential elevation of the head and torso using an automated device (ITD or ACD) [33]. The group did find, however, a higher likelihood of survival and neurological recovery when device therapy was initiated early [34].

## 5. Conclusions

Our study suggests an improved neurologic recovery with head elevation during extracorporeal circulation within the CARL therapy bundle following twenty minutes of untreated ventricular fibrillation in pigs. Within this complex translational research model, clinical follow-up revealed neurologic deficit patterns, which were not directly supported by histopathologic findings. Our results may serve as a basis to reconsidering traditional pathomechanisms and recovery potential in prolonged cardiac arrest.

## Figures and Tables

**Table 1:** Baseline characteristics. HUP: head-up, SUP: supine.

	HUP (*n* = 25)	SUP (*n* = 21)	
Male:Female	21:4	19:2	ns
Mean Body Weight (kg)	52.12 ± 3.71	54.40 ± 6.09	ns

**Table 2:** MAP and NSE. Mean arterial blood pressure (MAP) values in mmHg at baseline and after 15, 30, 45 and 60 min of controlled reperfusion. Neuron Specific Enolase (NSE) values in µg/L found at baseline, end of surgery, on postoperative day 1 and at the end of the experiment on day 7, immediately before euthanasia. HUP: head-up, SUP: supine. ns: not significant. Results are shown as mean +/− SD.

	HUP (*n* = 25)	SUP (*n* = 21)	
MAP (mmHg)			
Baseline	77.16 ± 13.60	81.30 ± 10.83	ns
15 min	110.11 ± 14.02	109.21 ± 15.56	ns
30 min	105.01 ± 16.92	104.19 ± 13.95	ns
45 min	107.08 ± 14.14	109.97 ± 8.60	ns
60 min	101.80 ± 17.38	109.85 ± 18.85	ns
NSE (µg/L)			
Baseline	0.78 ± 0.22	0.53 ± 0.17	ns
End of surgery	0.59 ± 0.17	0.46 ± 0.12	ns
Day 1	10.79 ± 2.33	6.73 ± 1.41	ns
Day 7	9.99 ± 8.20	10.81 ± 9.69	ns

**Table 3:** Hemoglobin, priming composition, neurologic recovery and survival. Data are presented as mean ± standard deviation. ^a^ *p* = 0.9077, ^b^ *p* = 0.001069, ^c^ *p* = 0.002414. HUP: head-up, SUP: supine. NDS [1,2,3,4,5,6,7]: neurologic deficit score [Days 1–7]. Favorable neurologic survival was defined as NDS < 50.

	HUP (*n* = 25)	SUP (*n* = 20)
Hemoglobin (g/dL)	9.28 ± 0.79	9.35 ± 0.89 ^a^
Sodium Priming (mmol/L)	146.24 ± 10.45	144.59 ± 9.61 ^b^
Osmolality of Priming (mosm/kg)	549.40 ± 20.06	524.39 ± 28.93 ^c^
Survival (days)	6.06 ± 1.64	4.10 ± 2.12
NDS1	108.96 ± 52.52	141.37 ± 61.79
NDS2	42.40 ± 31.66	88.29 ± 42.10
NDS3	26.80 ± 33.16	64.53 ± 43.54
NDS4	14.48 ± 17.80	33.33 ± 40.55
NDS5	12.38 ± 15.17	13.57 ± 21.99
NDS6	10.71 ± 13.30	6.67 ± 11.06
NDS7	10.24 ± 13.14	5.00 ± 11.18
Favourable Neurologic Survival	21	6

## Data Availability

Preliminary data to this publication have been presented at the European Resuscitation Council annual conference 2018 in Bologna, Italy. “Beneficial effects of head-elevation during Controlled Automated Reperfusion of the WhoLe Body (CARL) in the pig model”, Damjanovic, Domagoj et al., Resuscitation, Volume 130, e35 [35]. The data presented in this study are available on reasonable request from the corresponding author.

## Supplementary Materials

The following supporting information can be downloaded at: https://www.mdpi.com/article/10.3390/jcm12227054/s1, Supplement S1: ARRIVE checklist, Animal Research: Reporting In Vivo Experiments; Supplement S2: 2023_07_25_Heads-up-CARL; Supplement S3: Figure S1, Head positioning in the pig during the experiment.
