# Supplementary material for: The Impact of Head Position on Neurological and Histopathological Outcome Following Controlled Automated Reperfusion of the Whole Body (CARL) in a Pig Model
Source: J Clin Med. 2023 Nov 13;12(22):7054. doi: 10.3390/jcm12227054 (PMC10672538; doi:10.3390/jcm12227054)
Supplement: Supplementary file 1 [file jcm-12-07054-s001.zip › 2023_07_25_Heads-up-CARL_jcm-2299514_Supplement S2.pdf]

Supplement 2

Supplementary information

Calculations for Fig. 2

```
Linear mixed-effects model fit by maximum likelihood
  Data: nds_
      AIC      BIC    logLik
  2373.887 2394.696 -1180.944

Random effects:
 Formula: ~1 | pig
        (Intercept) Residual
StdDev:     29.8529 29.91607

Fixed effects:  nds ~ tag * HeadPos
               Value Std.Error  DF   t-value p-value
(Intercept) 28.22325 18.685605 191  1.510427  0.1326
tag         -5.20671  3.209175 191 -1.622445  0.1064
HeadPos     54.83227 12.558013  42  4.366318  0.0001
tag:HeadPos -6.40365  2.391413 191 -2.677768  0.0081
 Correlation:
            (Intr) tag    HeadPs
tag         -0.573
HeadPos     -0.944  0.569
tag:HeadPos  0.513 -0.944 -0.574

Standardized Within-Group Residuals:
        Min          Q1         Med          Q3         Max
-1.75505254 -0.60991388 -0.09954739  0.48872150  4.70809149

Number of Observations: 237
Number of Groups: 44
```

Calculations for Tab 2

```
    Kruskal-Wallis rank sum test

data:  HbStart by HeadPos
Kruskal-Wallis chi-squared = 0.00014071, df = 1, p-value = 0.9905


    Kruskal-Wallis rank sum test

data:  NaPriming by HeadPos
Kruskal-Wallis chi-squared = 0.038805, df = 1, p-value = 0.8438


    Kruskal-Wallis rank sum test

data:  OsmPriming by HeadPos
Kruskal-Wallis chi-squared = 8.8101, df = 1, p-value = 0.002996
```
