# Supplementary material for: The Impact of Head Position on Neurological and Histopathological Outcome Following Controlled Automated Reperfusion of the Whole Body (CARL) in a Pig Model
Source: J Clin Med. 2023 Nov 13;12(22):7054. doi: 10.3390/jcm12227054 (PMC10672538; doi:10.3390/jcm12227054)
Supplement: Supplementary file 1 [file jcm-12-07054-s001.zip › 2023_10_27_Heads-up-CARL_re-resubmission_Supplement S3.pdf]

Supplement 2

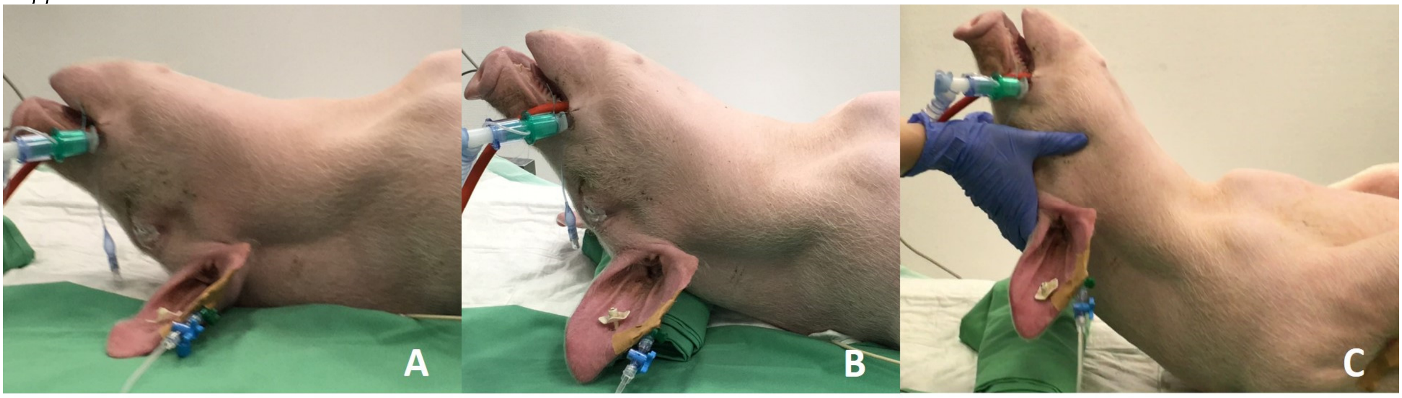

Supplementary Figure S1: Head positioning in the pig during the experiment. Panel A: Flat supine. Panel B: Head elevation using towel roll. Panel C: Additional manual inclination with further elevation. The inclination was performed intermittently.
