# Supplementary material for: The Impact of Head Position on Neurological and Histopathological Outcome Following Controlled Automated Reperfusion of the Whole Body (CARL) in a Pig Model
Source: J Clin Med. 2023 Nov 13;12(22):7054. doi: 10.3390/jcm12227054 (PMC10672538; doi:10.3390/jcm12227054)
Supplement: Supplementary file 1 [file jcm-12-07054-s001.zip › ARRIVE checklist Heads Up CARL_Supplement S1.pdf]

ARRIVE checklist

Animal Research: Reporting *In Vivo* Experiments

	Item	Remarks
**TITLE**	**Title**<br>**L 1-4**	**The impact of head position on neurological and histopathological outcome following Controlled Automated Reperfusion of the whoLe Body (CARL) in a pig model**
**ABSTRACT**	**Abstract**<br>**L31-52**	**Introduction:** Based on extracorporeal circulation, targeted reperfusion strategies have been developed to improve survival and neurologic recovery in refractory cardiac arrest: Controlled Automated Reperfusion of the wholLe Body (CARL). Furthermore, animal and human cadaver studies have shown beneficial effects on cerebral pressure due to head elevation during conventional cardiopulmonary resuscitation. Our aim was to evaluate the impact of head elevation on survival, neurologic recovery and histopathologic outcome in addition to CARL in an animal model. **Methods:** After 20 minutes of ventricular fibrillation, 46 domestic pigs underwent CARL, including: High, pulsatile extracorporeal blood flow, pH-stat-acid-base-management, priming with a colloid, mannitol and citrate, targeted oxygen, carbon dioxide and blood pressure management, rapid cooling and slow rewarming. N=25 were head-up, HUP during CARL, and N=21 were flat supine, SUP. After weaning from ECC, the pigs were extubated and followed up in the animal care facility for up to seven days. Neuronal density was evaluated in neurohistopathology. **Results:** More animals in the HUP group survived and achieved a favorable neurological recovery, 21/25 (84%) versus 6/21 (29%) in the SUP group. Head positioning was an independent factor in neurologically favorable survival ($p<0.00012$). Neurohistopathology showed no significant structural differences between HUP and SUP. Distinct, partly transient clinical neurologic deficits were blindness and ataxia. **Conclusion:** Head-elevation during CARL after 20 minutes of cardiac arrest independently improved survival and neurologic outcome in pigs. Clinical follow-up revealed transient neurologic deficits potentially attributable to functions localized

		in the posterior perfusion area, whereas histopathologic findings did not show corresponding differences between the groups. A possible explanation of our findings may be venous congestion and edema as modifiable contributing factors of neurologic injury following prolonged cardiac arrest.<br>Key words: ischemia-reperfusion; extracorporeal life support; venous return; porcine cardiac arrest model; bundle of care; targeted CPR; eCPR; va-ECMO; pathophysiology of cardiac arrest
**INTRODUCTION**		
- **Background**	**L 56-81**	One of the main therapeutic targets in post cardiac arrest care is the mitigation of ischemia-reperfusion-injury, IRI. Controlled Automated Reperfusion of the whoLe body, CARL is an individualized therapy bundle based on extracorporeal circulation and designed to reduce IRI. With CARL, favourable neurologic recovery could be achieved in our porcine cardiac arrest model, even after 20 minutes of normothermic cardiac arrest.<br><br>Elevated positioning of the head is an established intervention applied for prophylaxis and therapy of increased intracranial pressure, ICP. During conventional CPR, elevation of the head was shown to reduce ICP in a porcine cardiac arrest model.
- **Objectives**	**L82-83**	To evaluate the impact of head elevation on survival, neurologic recovery and neuronal loss, in combination with CARL therapy
**METHODS**		
- **Ethical statement**	**L 85-88**	Animal experiments were approved by the local ethics committee (Freiburg, Germany, approval number G-15/148) and performed in accordance with the rules and regulations of the German animal protection law and the animal care guidelines of the European Community (2010/63/EU).
- **Study design**	**L89-92**	The basic protocol of this chronic porcine cardiac arrest model has already been published in detail.<br><br>Taunyane et. al.:<br>Anaesthesia, monitoring and blood sampling

			All animals were premedicated with ketamine and midazolam intramuscularly before induction of anaesthesia. An intravenous catheter was inserted into the marginal ear vein for induction of anaesthesia with propofol and muscle relaxation with vecuronium. Endotracheal intubation was performed with a size 7.0 mm endotracheal tube. Volume-controlled mechanical ventilation was provided to normalize pH, PaO2 and PaCO2. Anaesthesia was maintained with inhaled isoflurane (1.5–2%) and infusions of vecuronium (0.2 mg/kg/h) as well as fentanyl (2 µg/kg/h). Antibiotic prophylaxis was provided with ceftriaxone. Continuous non-invasive monitoring of the electrocardiogram, arterial saturations (Siemens, SC 9000XL, MA, USA) and electroencephalogram were documented. A 7-Fr pulmonary artery catheter (Arrow Thermodilution Balloon Catheter, PA, USA) was inserted into the right subclavian vein to monitor right heart pressures, pulmonary artery wedge pressures and cardiac output continuously by the thermodilution method. The neck vessels were exposed through a right paratracheal surgical dissection. After vessel preparation, we cannulated (20G Vygon Arterial Leader Cath, Ecouen, France) the right common carotid artery in a caudal direction to obtain continuous arterial blood pressure monitoring and blood sampling for arterial blood gas analysis. The right internal jugular vein was cannulated in a cranial direction to obtain blood samples for analysis.<br><br>This study was conducted as pooled analysis. Experiments were carried out in an open-label parallel-group design. The group allocation was not randomized, but followed convenience sampling.
-	**Experimental procedures**	**L93-146**	Pigs were anesthetized, and ventricular fibrillation was induced. After 20 minutes of

			normothermic CA, CARL was started. It comprises a targeted reperfusion therapy bundle, based on arteriovenous extracorporeal membrane oxygenation. This, in brief, consisted of high, pulsatile extracorporeal blood flow, pH-stat-acid-base-management, priming with a colloid (20% human albumin [Albiomin 200 g/l, Biotest Pharma GmbH, Dreieich, Germany or 200 g/l, Baxalta Deutschland GmbH, Unterschleissheim, Germany or 200 g/l, Octapharma GmbH, Langenfeld, Germany respectively, depending on availability] or Gelatin-Polysuccinate, [Gelafundin 40 g/l, B. Braun Melsungen AG, Melsungen, Germany]), mannitol and citrate, tight oxygen and carbon dioxide control, rapid cooling and slow rewarming before weaning off extracorporeal circulation, ECC. Instead of electric defibrillation, ventricular fibrillation was terminated via cardioplegic potassium bolus preferably.<br><br>Head elevation was conducted using a standardized pillow, with additional regular inclinations for up to 90°.
-	**Experimental animals**	**L93**	Juvenile domestic German landrace pigs.
-	**Housing and husbandry**	**L117-131**	Animals were accomodated at the experimental facility *CEMT* (Center for Experimental Models and Transgenic Service, University Medical Center Freiburg) 1-2 days prior to the experiment. They were fasted overnight, with free access to water.<br><br>After the intervention, they received usual care at the CEMT by a veterinarian and trained staff. Monitoring during the first few hours comprised pulsoximetry and supplemental oxygen administration as needed. No advanced cardiorespiratory monitoring was used. Fluids and medication (analgesia, sedation, anticonvulsants) were administered intravenously until PO drinking and eating was possible. Physiotherapy, mobilization, clinical evaluation and scoring were conducted by the veterinarian and trained staff. Usually on postoperative day 2 or 3, enrichment (straw) was added.

-	**Sample size**	**L114**	N=46
-	**Allocating animals to experimental groups**	**L90-92, L114-116**	The group allocation was not randomized, but experiments were carried out blockwise.
-	**Experimental outcomes**	**L122-146**	Neurologic outcome was characterized using a species-specific Neurologic Deficit Score. A score of 500 denotes brain death, whereas animals with NDS < 50 were able to stand up independently, walk without help and eat on their own. A score <50 was therefore defined as a surrogate of favorable neurologic outcome, or significant potential for rehabilitation, respectively. Upon clinical suspicion, distinct neurologic deficits were evaluated separately. After completion of clinical follow-up and euthanasia, the porcine brains were retrieved for neuro-histopathologic workup, for which hematoxylin-eosin-staining was used. For this purpose, a frontal craniotomy was performed. After transsecting at the level of the medulla oblongata, brains were extracted as a whole and immediately immersed in formaldehyde 4%. Following one week of immersion, the brains were dissected and 5mm samples of the frontal lobe, cerebellar vermis and hippocampus were obtained, respectively. In identifying those areas as our regions of interest, we sought to procure a representation of both the territories of the anterior and occipital cerebral blood supply, respectively. Furthermore, the hippocampal region has been identified as one of the most susceptible regions for cerebral ischemia-reperfusion injury following cardiac arrest[17]. In the next step the samples were dehydrated and embedded in paraffin. Using a microtome, 3µm microtome slices of the samples underwent hematoxylin-eosin-staining. Consequently, slides were masked, digitalized, and representative areas were examined in terms of neuron count to determine neuron density. Only neurons with a clearly identifiable nucleus were considered to be viable and have been included in the total count.
-	**Statistical methods**	**L147-156**	Continuous baseline characteristics were compared between the groups (HUP or SUP) using the Kruskal-Wallis rank sum test.

		Kaplan-Meier survival curves were calculated to compare survival up to 7 days between the groups. Regarding NDS, linear mixed models (see Brown and Prescott, 1999) have been fitted with a random intercept (subject=pig). The continuous response variable "NDS" is modelled as a linear function of time (continuous) and group (HUP or SUP), including an interaction term. We further included all baseline characteristics if they show significant variations between the groups. All computations were performed using the statistical software R system. They are added as Supplement 2
**RESULTS**		
- **Baseline data**	**Results**<br>**Table 1**<br>**L158**	See table 1.
- **Numbers analyzed**	**Results 184**<br>**Figure 2, Figure 3, Figure Legend**	45/46 pigs were successfully weaned from ECC after 1 hour of CARL.
- **Outcomes and estimation**	**Binary outcome.**<br>**Figure 2-4**<br>**Figure Legend**	See figures.<br><br>Animals in the heads-up group showed significantly better neurological recovery (NDS<50) compared to supine positioning, 21/25 (84%) vs 6/21 (29%).<br><br>During clinical follow-up, predominant clinical problems were transient blindness and ataxia, in otherwise alert animals, most of whom started eating and drinking early in the postoperative course.<br><br>After adjusting for different confounders, head positioning remained an independent factor in neurologically favorable survival (p<0,00012).
- **Adverse events**	**Results Paragraph 3, L185**	1 animal could not be weaned off ECC due to refractory haemodynamic failure.<br><br>Immediately postoperatively, supplemental oxygen was required in the majority of animals.<br><br>Seizures and status epilepticus usually occurred during the first 24 hours, responding to sedatives (midazolam) and/ or

			anticonvulsants (levetiratecam), and wearing off following postoperative day 1.<br><br>During clinical follow-up, predominant clinical problems were transient blindness and ataxia, in otherwise alert animals.
**DISCUSSION**			
-	**Interpretation/scientific implications**	**Discussion L224-343**	See discussion.
-	**Generalisability/translation**	**Discussion L 344-359**	See discussion.
-	**Funding**	**Acknowledgements section, Funding statement.**	
